# High-Moisture Meat Analogues Produced from Yellow Pea and Faba Bean Protein Isolates/Concentrate: Effect of Raw Material Composition and Extrusion Parameters on Texture Properties

**DOI:** 10.3390/foods10040843

**Published:** 2021-04-13

**Authors:** Ferawati Ferawati, Izalin Zahari, Malin Barman, Mohammed Hefni, Cecilia Ahlström, Cornelia Witthöft, Karolina Östbring

**Affiliations:** 1 Department of Chemistry and Biomedical Sciences, Linnaeus University, 39231 Kalmar, Sweden; mohammed.hefni@lnu.se (M.H.); cornelia.witthoft@lnu.se (C.W.); 2 Department of Food Technology, Engineering and Nutrition, Lund University, 22362 Lund, Sweden; izalin.zahari@food.lth.se (I.Z.); cecilia.ahlstrom@food.lth.se (C.A.); karolina.ostbring@food.lth.se (K.Ö.); 3 Department of Biology and Biological Engineering, Chalmers University, 41296 Gothenburg, Sweden; malin.barman@chalmers.se; 4 Food Industries Department, Faculty of Agriculture, Mansoura University, Mansoura 35516, Egypt

**Keywords:** high-moisture extrusion (HME), plant protein, meat analogue, yellow pea, faba bean, pulses

## Abstract

Yellow pea and faba bean are potential candidates to replace soybean-based ingredients due to their suitability for cultivation in the northern hemisphere, non-genetically modified organisms cultivation practice and low risk of allergenicity. This study examined the functionality of local yellow pea and faba bean protein isolates/concentrate as meat analogue products. The most critical factors affecting the texture properties of meat analogue were also determined. Extrusion was used to produce high-moisture meat analogues (HMMAs) from yellow pea and faba bean protein isolates/concentrates and HMMAs with fibrous layered structures was successfully produced from both imported commercial and local sources. The texture properties of the HMMA produced were mainly affected by the ash, fiber and protein content and water-holding capacity of the source protein. Three extrusion process parameters (target moisture content, extrusion temperature, screw speed), also significantly affected HMMA texture. In conclusion, functional HMMA can be produced using protein isolates derived from locally grown pulses.

## 1. Introduction

High consumption of resource-intensive foods, such as animal-based products, is associated with high greenhouse gas emissions, placing a heavy burden on the food system [1]. Transition to a more sustainable diet with higher intake of plant-based foods has been identified as a key factor in improving health and reducing environmental pressure on the current food system [2,3]. Pulses are considered one of the most promising plant-based raw materials in this transition. However, consumption of locally grown pulses is low in Sweden, probably due to lack of attractive products and to anti-nutritional factors in pulses interfering with nutrient absorption [4]. There is a fast-growing market for extruded products, such as meat analogues, and extrusion technology is expected to provide various pulse-based food products that meet consumer acceptability and increase consumption of pulses in Western society.

Despite high demand for plant-based meat analogues among consumers, the texture properties remain a challenge [5,6]. Meat analogues can be produced using low- and high-moisture extrusion cooking. Products prepared by the low-moisture extrusion technique have a porous structure and the texture does not resemble animal flesh [7]. In contrast, high-moisture extrusion (HME) can create a product with a fibrous meat-like structure, known as a high-moisture meat analogue (HMMA), from plant protein raw materials [7,8]. During the HME process, plant proteins are unfolded, aggregated and realigned with heat, pressure and shear in the extruder barrel [7]. In the cooling die, the protein molecules are cross-linked, leading to the formation of a fibrous meat-like structure [7]. The covalent disulphide bonds and, to a smaller extent, the non-covalent bonds between proteins are suggested to be essential in forming the fibrous structure of HMMAs [9]. The fibrous structure formation also depends on several other factors, among which the amount and type of proteins in the raw material are important. Empirical evidence suggests that there is a critical limit for protein concentration in the raw material, with protein concentrations below the limit severely affecting the possibility of creating a fibrous texture in the extrudate. This critical limit is reported to be around 50%, which means that protein concentrates or isolates (50–90% protein content) are needed for formation of the layered fibrous structure in HMMAs [10,11].

Soy protein is by far the most studied plant protein in extrusion cooking, due to its techno-functionality and nutritional properties [12,13,14,15,16,17,18,19]. There are also disadvantages to soybeans, such as its allergenic potential and cultivation using genetically modified organisms (GMOs) [8]. Soybean is also unsuitable for cropping in the northern hemisphere and has to be imported, which could reduce the potential environmental sustainability of associated plant-based products. As an alternative to soybean, locally grown pulses are of particular interest due to the high protein content, overall low potential for allergens and non-GMO cultivation [11]. Pulses are also reported to have promising techno-functional properties [11]. In Sweden, pulse cropping is dominated by faba bean and yellow pea. These crops are currently mostly used in animal feed, although there is increasing interest in utilizing faba bean and yellow pea for food production [20].

To our knowledge, few works have been published on HME using pea protein isolate [8,9,21,22] and only one on HME using faba bean protein concentrate [22]. Hence, the main objectives of the present study were to (1) study the use of protein isolates from locally grown yellow pea and faba bean as compared to commercial protein ingredients for production of HMMA; and (2) evaluate the effects of raw material composition and extrusion parameter settings on HMMA texture.

## 2. Materials and Methods

### 2.1. Materials

Two commercial protein ingredients were used in the study: commercial yellow pea protein isolate (79% protein on a wet basis (wb), imported from Vancouver, Canada) was purchased from Bulk Powders Company, Colchester, UK, and commercial faba bean protein concentrate (Faba Protein F65X, 56% protein wb, imported from Tau, Norway) was purchased from Vestkorn A/S, Tau, Norway. For protein isolate preparation in the main experiments, locally grown yellow pea and faba bean grains (harvested in 2019) were obtained from Kalmar-Ölands Trädgårdsprodukter, Färjestaden, Sweden. Sodium hydroxide (NaOH) and citric acid powder were obtained from VWR International, Stockholm, Sweden. Raw chicken breast, beef, and commercial HMMA made from soybean (Oumph, Food for Progress Scandinavia AB, Stockholm, Sweden), purchased at a local supermarket in Lund, Sweden, were used as references. Chicken and beef were chosen as references because they are the most common animal-based protein sources, whereas Oumph is the only commercial meat analogue produced using HME technique available in Sweden as of now. Before texture analysis, the chicken breast and beef were cut and boiled for 15 min and cooled, while the commercial HMMA was thawed and brought to room temperature.

### 2.2. Preparation of Protein Isolates from Pulses

Protein isolates from yellow pea and faba bean were produced in a small pilot-scale plant, using alkaline extraction followed by isoelectric precipitation. Pulse seeds (3 kg) were milled to flour using a laboratory mill (Laboratory Mill 120, Perten Instruments AB, Hägersten, Sweden). The pulse flour obtained was mixed with water (1:9 *w*/*w*) and the pH was adjusted to 10.5 with 2M NaOH. The dispersion was stirred for 1 h at 350–400 rpm (IKA RW 28 digital, Germany), with the pH re-adjusted to pH 10.5 every 10 min. Following this leaching step, the dispersion was separated in a decanter using a 56 mm weir disc (Decanter centrifuge DM80, Lemitech GMBH, Berlin, Germany) at an acceleration of 2000 g and differential force of 10 rpm. The influx flow was set to 20 L/h with a peristaltic pump (Masterflex Easy-load Model 77200-62, Cole-Parmer, Vernon Hills, IL, USA). The spent solids phase was discarded. The light liquid phase containing the majority of the proteins was collected and the pH was adjusted to 5.0 with citric acid powder, to precipitate the proteins. The light liquid phase was then centrifuged (Allegra X-15R Centrifuge, Beckman Coulter, Brea, CA, USA) at 4700 g at 20 °C for 20 min. The precipitate was collected, neutralized to pH 7 with 2M NaOH and freeze-dried (Epsilon I/30, Martin Christ Gefriertrocknungsanlagen GmbH, Osterode, Germany). Each protein isolate was photographed to visualize the color. The yellow pea and faba bean protein isolates, which are referred to hereafter as ‘YPI-local’ and ‘FBI-local’, respectively, were kept at −18 °C until further experiments and analyses.

### 2.3. Characterisation of Commercial and Local Protein Isolates/Concentrate

#### 2.3.1. Proximate Analysis

The crude protein content of commercial and local yellow pea and local faba bean isolates/concentrate was determined using a protein analyser (Flash EA 1112 Series, Thermo Scientific, Waltham, MA, USA) according to the Dumas combustion method AOAC 990.03 [23]. A conversion factor of 6.25 was used to calculate total protein content. Total fat content was determined by solvent extraction in a semi-automatic Soxtec apparatus (Tecator AB, Höganäs, Sweden), using petroleum ether as solvent according to AOAC 920.39 [24]. Total dietary fiber (TDF) content was determined using the K-TDFR kit (Megazyme, Bray, Ireland), according to AOAC 991.43 [25]. Moisture content was determined by oven-drying the protein isolate/concentrate samples at 105 °C for 16 h, according to AOAC 934.01 [26]. For ash content determination, samples were transferred to porcelain crucibles and incinerated in a furnace at 550 °C for 16 h, according to AOAC 923.03 [27]. Carbohydrate content was calculated by difference. All analyses were performed in triplicate.

#### 2.3.2. Thermal Properties

The thermal properties of the protein isolates/concentrate were determined according to Zahari et al. [28], using a differential scanning calorimetry instrument (EXSTAR6000 DSC, Seiko Instruments Inc., Shizuoka, Japan. Approximately 2 mg of protein isolate/concentrate were weighed into an aluminum pan and MilliQ water (three times sample weight) was added. The pan was sealed and heated from 25 °C to 160 °C at a rate of 10 °C/min. The denaturation temperature (T_d_) and enthalpy of denaturation (ΔH) were recorded. Triplicate measurements were carried out for each raw material.

#### 2.3.3. Water-Holding Capacity

Water-holding capacity (WHC) of the protein isolates/concentrate was measured according to the AACC method 56–30.01 [29], on 5 g portions of protein isolate/concentrate mixed with 25 g distilled water and centrifuged at 2000 g for 10 min. WHC was expressed as grams of water retained per gram of powder.

### 2.4. High-Moisture Extrusion Trials

**Figure 1:**
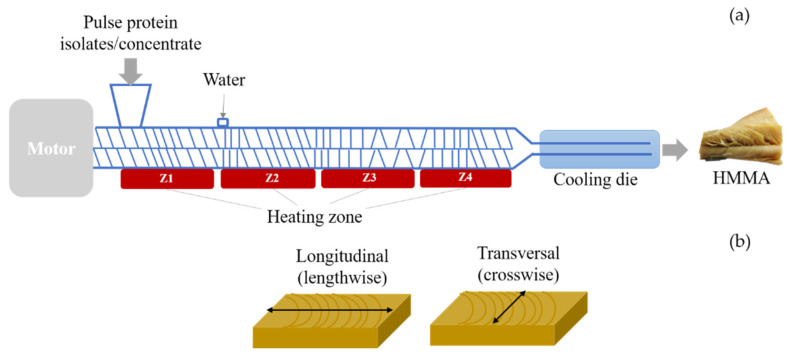
(**a**) Schematic diagram of a twin-screw extruder and cooling die, modified illustration adopted from Maung & Ryu [7] and (**b**) cutting directions on high-moisture meat analogue (HMMA) in cutting strength measurements.

**Figure A1:**
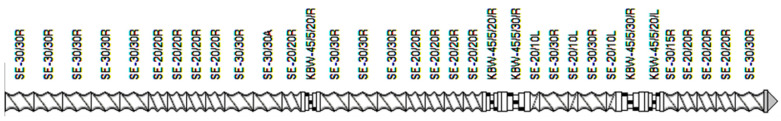
Screw configuration (from start (left) to end) used for high-moisture meat analogue (HMMA) extrusion (KETSE 20/40D extruder, Brabender GmbH and Co., Duisburg, Germany). SE = screw element, KBW = kneading block.

All extrusion experiments were performed using a laboratory co-rotating twin-screw KETSE 20/40D extruder (Brabender GmbH and Co., Duisburg, Germany) with a fixed screw configuration (Appendix A, Figure A1). Screw diameter (D) was 20 mm and the screw length/diameter ratio was 40:1. The extruder barrel was segmented into four temperature-controlled zones (Z1–Z4) (Figure 1a), heated by an electric cartridge heating system and cooled with running water. A cooling die (7 mm × 25 mm × 300 mm) was attached to the end of the extruder, with water as the cooling medium (20 °C), to prevent expansion and facilitate fibrous texture formation. The protein isolate/concentrate and water feed rate were calculated according to the initial moisture content of each protein isolate/concentrate, the target moisture content for HMMA and total mass flow. HMMA samples were kept in a sealed plastic bag and analyzed for texture parameters on the same day as the extruder trials.

Due to the limited availability of protein isolates from local pulses, preliminary trials using commercial yellow pea protein isolate (YPI-com) were conducted to define the experimental settings for the extrusion parameters. In the first preliminary trial, to determine the extrusion temperature range, different temperatures above the denaturation temperature of the isolate were explored with the focus on heating zones 3 and 4 (Z3 and Z4) (Appendix B, Table A1). Thereafter, different levels of target moisture content (66–70%) and screw speed (400, 600, 800 rpm) were investigated (Appendix B, Table A3), until fibrous structures were formed in the extruded material exiting the cooling die. From the pre-study, a target moisture content below 66% resulted in jammed and burnt materials in the extruder, whereas a target moisture content above 70% did not result in a compact product. Selected extrusion parameters were also varied for the commercial faba bean protein concentrate (FBC-com) (Appendix B, Table A3). The HMMA samples from each trial stage were subjected to texture analysis (below) and compared with the reference products (boiled chicken breast, boiled beef, and commercial HMMA from soybean). The best combination of target moisture content, screw speed and extrusion temperature that resulted in a fibrous structure was then used to produce HMMA from the protein isolates from local yellow pea and faba bean (YPI-local, FBI-local) (Appendix B, Table A3). Specific mechanical energy (SME, kJ/kg) was calculated using the formula [30]:

(1) $$\mathrm{SME}=\frac{2\pi \;\times \;n\;\times \;T}{\mathrm{MFR}}$$

where *n* is the screw speed (400, 600, 800 rpm), *T* is torque (obtained from % drive load × 80 Nm) and MFR is mass flow rate (2 kg/h).

#### Texture Properties of HMMA

Texture profile analysis (TPA) of the HMMA obtained was carried out in triplicate, using a texture analyzer (TVT-300XP, Perten Instruments AB, Hägersten, Sweden), according to Zahari et al. (2020) [28]. The meat analogue samples were cut and shaped into 20 mm × 20 mm pieces (7 mm thickness), and then compressed by 2 mm from initial height using a cylindrical probe (diameter 18 mm). The hardness, springiness and chewiness of the samples were recorded. A second analysis was carried out with a cutting test used previously to assess fibrous structure formation in meat analogues [8,16]. The cutting strength was evaluated using a knife blade (height 117 mm) penetrating the sample (20 mm × 20 mm, 7 mm thickness) to 5 mm depth at a speed of 2 mm/s. Triplicate samples were cut longitudinally (lengthwise) and transverse (crosswise) to the outflow direction from the extruder, as depicted in Figure 1b. Similar values in longitudinal and transverse cuts indicate that the meat analogue sample has a uniform texture, with no fibrous layered structure [8].

### 2.5. Statistical Analysis

All data were expressed as mean ± standard deviation (SD) (*n* = 3). One-way ANOVA and post-hoc Tukey’s test were used to determine significant differences in proximate composition and WHC between the different protein isolates/concentrate. The level of significance was set to 0.05. ANOVA analyses were performed using Graphpad Prism software version 7.04-2017. Multivariate analysis, principal component analysis (PCA) and partial least square analysis (PLS) were performed using Simca software version 16.0.1. PCA was used to identify similarities or groupings in the data and to determine how the protein isolates/concentrate composition and extrusion parameters correlated to the texture properties of the HMMA. PLS was used as a supervised technique to identify the correlation between protein isolate/concentrate composition, extrusion parameters (as input X) and HMMA texture properties (as output Y). The orthogonal partial least squares (OPLS) model was used to determine the correlation between input X to one specific texture parameter (Y). The most important factors affecting HMMA texture were determined from the variable of importance (VIP ≥ 1) plot.

## 3. Results and Discussion

### 3.1. Characterisation of Protein Isolates/Concentrate from Pulses

**Figure A2:**
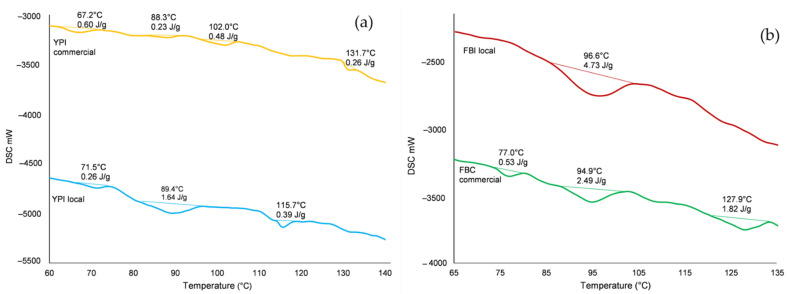
Denaturation temperature and enthalpy of (**a**) yellow pea isolate and (**b**) faba bean isolate.

There were significant differences (*p* < 0.05) in proximate composition between the protein isolates/concentrate. Commercial faba bean concentrate (FBC-com) had the lowest protein content of all samples (56% wb), while local faba bean isolate (FBI-local) had the highest (88% wb) (Table 1). This was because FBC-com was produced using a dry fractionation technique that gave lower protein purity than the wet protein isolation technique [31] used for the local pulses and the commercial yellow pea protein isolate (YPI-com). Moreover, FBC-com had the highest carbohydrate and fiber content also due to the fractionation method used. Although FBC-com was prepared from dehulled grains, the fiber content was very high as compared with the other samples (Table 1). Insoluble fiber should have been removed during dehulling. However, the soluble fiber fraction might not completely detach from the protein, leading to a high fiber content in the dry-fractionated protein [32]. In contrast, the starch and fiber fractions were separated in two stages during the wet isolation technique [31]. The fat content of YPI-com and FBI-local was negligible, whereas YPI-local and FBC-com had a fat content around 3% wb (Table 1). The high fat content in FBC-com might be due to incomplete separation of fat from the protein fractions during dry-fractionation [33]. As YPI-local and FBI-local were prepared using the same method, the difference in lipid retention during the extraction might be due to different lipid composition and location in the grain matrix [34]. Different numbers of endothermic peaks were observed for yellow pea and faba bean proteins (Appendix B, Figure A2), four peaks identified in YPI-com (67 °C, 88 °C, 102 °C, 132 °C) and three in YPI-local (72 °C, 89 °C, 116 °C) (Appendix B, Figure A2). Furthermore, FBC-com had three endothermic peaks (77 °C, 95 °C, 128 °C), whereas FBI-local only had one peak at 97 °C (Appendix B, Figure A2). The first endothermic peak for both yellow pea and faba bean proteins is assumed to be the starch gelatinization peak, as observed by doCarmo et al. [32]. The main endothermic peak at 88–89 °C for yellow pea and at 95–97 °C for faba bean might correspond to the denaturation temperature of globulins which are the major storage proteins in pulses [32,35]. The highest melting temperature was 132 °C for yellow pea protein and 128 °C for faba bean protein. This indicates that the cooking temperature zone in the extruder should be set above 132 °C to achieve a complete melt of the food mix which is critical for the production of meat analogue products [8].

The YPI-com, YPI-local and FBI-local had similar WHC (3 mL/g), which were around three-fold higher than that of FBC-com (1 mL/g) (Table 1). According to Lam et al. [36], proteins are more denatured during the wet isolation process, leading to changes in tertiary and quaternary protein structure. These structural changes may increase exposure of hydrophilic groups, in turn increasing the ability to hold water. WHC is an important functional property in food formulation, due to the effect on the texture of the final product [37].

**Figure 2:**
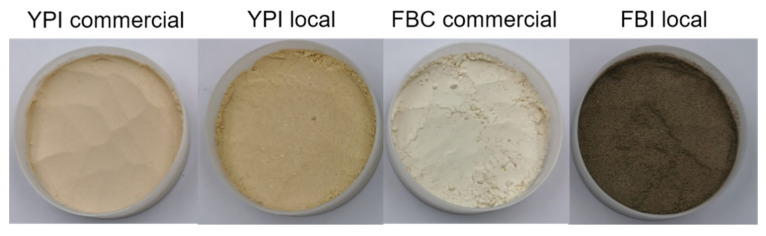
Appearance of the different types of pulse protein isolates/concentrate. YPI = yellow pea isolate, FBC = faba bean concentrate, FBI = faba bean isolate.

In terms of color, FBI-local was dark grey, whereas the commercial protein concentrate was light-colored (Figure 2). According to information from the supplier, FBC-com was made using dehulled faba bean grains as the raw material, whereas for FBI-local the whole faba bean grains, with the hull intact, were used. It was observed in the leaching phase that the color of the dispersion gradually became darker over time during the incubation for 1 h. Based on Sharan et al. [35], this could be due to the content of proanthocyanidins in faba bean hull being subjected to oxidation. The color of the FBI-local protein precipitate after pH adjustment with acid was light grey, but it changed to dark grey when the precipitate was neutralized (pH 7) just before freeze-drying. Thus, the precipitate could be freeze-dried directly after acidification to avoid dark coloration of the protein isolate, although there is the risk of an acidic flavor if the pH is not neutralized.

### 3.2. High-Moisture Extrusion Trials

#### 3.2.1. Preliminary Extrusion Trials

In preliminary trials using YPI-com to define the extrusion parameter settings (extrusion temperature, screw speed, target moisture content), the temperature in the four heating zones (Z1–Z4) in the extruder barrel was adjusted individually. The temperature in Z1 and Z2 was kept at 40 and 80 °C, respectively, while in Z3 and Z4 four combinations of extrusion temperatures ranging from 110–150 °C were tested (Appendix B, Table A1).

The texture of the extrudate was affected by the temperature in Z3 and Z4, with higher temperature giving extrudates with reduced hardness and chewiness. However, there was no apparent effect of extrusion temperatures on the cutting strength of HMMA (Appendix B, Table A2). At an extrusion temperature of 110–110 °C in Z3–Z4, the samples were compact, but no fibrous structures were formed. With increased extrusion temperature in Z3–Z4, fibrous layered structures started to form and were most evident at 130–150 °C on visual inspection. Others have observed a similar effect of extrusion temperature on fiber formation, in extrudates produced from pea and soy protein, respectively [8,38]. It has been suggested that, in order to obtain layered fiber in extrudates, the food mix (e.g., protein, carbohydrate, water) has to be completely melted in the extruder barrel [8,38]. At too low extrusion temperature, the food mix is only partly melted and non-melted molecules may disperse in the matrix, causing poor layered fibrous formation. An increased extrusion temperature will induce complete melting of the food mix, with reduced viscosity and initiation of layered fibrous structure formation. The texture of the extrudates was also affected by the target moisture content, with a higher moisture content giving a softer extrudate (Appendix B, Table A2).

A decrease in specific mechanical energy (SME) was observed as the extrusion temperature increased (Appendix B, Table A2), confirming findings by others [30,39]. This was due to lower viscosity of the material in the barrel as a result of higher extrusion temperature [30]. SME reflects the mechanical work put into the material during extrusion [33] and can affect final product characteristics such as hardness, density and expansion index [16].

Differential scanning calorimetry (DSC) analysis of YPI-com indicated that its highest melting temperature was 132 °C (Appendix B, Figure A2). To achieve complete melting of the food mix and facilitate fibrous structure formation in the HMMA, the extruder should therefore be operated above 132 °C when processing yellow pea protein isolate. The chosen extrusion temperature profile for YPI-com was 40-80-130-150 °C (Z1-Z2-Z3-Z4). This temperature profile was also used to produce HMMA from FBC-com, but a minor adjustment was needed in Z2 to prevent backflow during extrusion. Thus, the chosen extrusion temperature profile for FBC-com (Z1-Z2-Z3-Z4) was 40-60-130-150 °C.

From the preliminary trials, it was concluded that the target moisture content to produce HMMA from YPI-com was around 68% with extrusion temperature 40-80-130-150 °C (Z1-Z2-Z3-Z4), whereas HMMA made from FBC-com required moisture content around 60–62% with extrusion temperature 40-60-130-150 °C (Z1-Z2-Z3-Z4) in order to form a fibrous structure and to achieve similar texture properties as the reference materials.

#### 3.2.2. HMMA Production Using Local Pulse Protein Isolates

**Figure 3:**
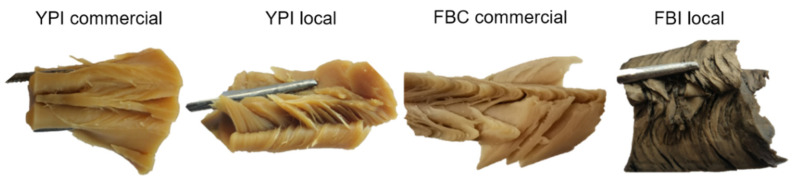
High-moisture meat analogues (HMMAs) produced from different pulse protein isolates/concentrate. YPI = yellow pea isolate, FBC = faba bean concentrate, FBI = faba bean isolate.

In terms of protein content, YPI-local contained 81% protein, which was similar to the level in YPI-com (79%) (Table 1). Thus, production of HMMA from YPI-local followed the extrusion parameters for YPC-com. HMMA samples with prominent layered fibrous structure (Figure 3) were successfully produced from YPI-local at: 67% target moisture content, extrusion temperature 40-80-130-150 °C (Z1-Z2-Z3-Z4) and screw speed 400 and 600 rpm (Appendix C, Table A4). No sample was obtained at screw speed 800 rpm, due to unstable flow of output.

For FBI-local, the extrusion parameters used for YPI-com and FBC-com were initially applied. However, these settings led to backflow and jammed material in the cooling die, so the temperature in Z3 and Z4 had to be decreased to allow consistent flow. HMMA with layered fibrous structure (Figure 3) was successfully produced from FBI-local at: 62% target moisture content, extrusion temperature 40-60-110-130 °C (Z1-Z2-Z3-Z4) and screw speed 800 rpm (Appendix C, Table A4). Proteins with low denaturation temperature do not require high extrusion temperature and can easily be denatured and texturized by pressure and shear in the extruder [17]. FBI-local had a protein denaturation peak at 97 °C (Appendix B, Figure A2) which might explain why a fibrous structure formed even though the extrusion temperature was lower. Thus, the runs confirmed that HMMA could be produced from yellow pea and faba bean proteins (both commercial and local). Some modifications were needed in the temperature zones, screw speed and target moisture content to create a layered fibrous structure and ensure a consistent flow of extrudates.

The HMMA produced from yellow pea and faba bean was significantly (*p* < 0.05) harder compared to the reference commercial soybean HMMA, but similar to the boiled chicken and beef depending on extruder settings (Appendix C, Table A4). The springiness did not differ (*p* > 0.05) between the HMMA produced and the references, independent on origin. The commercial soybean HMMA had significantly (*p* < 0.05) lower chewiness than any of the HMMA produced as well as the chicken and beef reference. The HMMA produced from yellow pea had similar chewiness as the boiled chicken and beef, depending on the extruder setting. In contrast, the HMMA produced from faba bean, independent of the extruder settings, was significantly chewier than the references.

HMMA produced from YPI-com, YPI-local and FBC-com had higher crosswise than lengthwise cutting strength (Appendix C, Table A4). This indicates that the fibers were more textured in the longitudinal direction. In contrast, HMMA made from FBI-local had higher lengthwise than crosswise cutting strength (Appendix C, Table A4). Hence, the fibers in FBI-local were more aligned in the crosswise direction (parabolic pattern), resembling those in the reference samples (boiled chicken, boiled beef, commercial soy HMMA). In extrusion of HMMA from pea protein with 55% moisture content at 130 °C, Osen et al. [8] also observed layered fibrous structures in parabolic patterns. Upon raising the extrusion temperature to 160 °C, they found that a predominantly lengthwise fibrous structure appeared [8], as also observed in the present study. Osen et al. [8] suggested that the melt viscosity decreases as the extrusion temperature increases, affecting the flow pattern in the cooling die. The higher temperature leads to higher flow velocity at the core of the flow profile, which upon solidification might lead to more lengthwise-oriented fibers [8].

### 3.3. Effect of Protein Isolate/Concentrate Composition and Extrusion Parameters on HMMA Texture Properties

**Figure 4:**
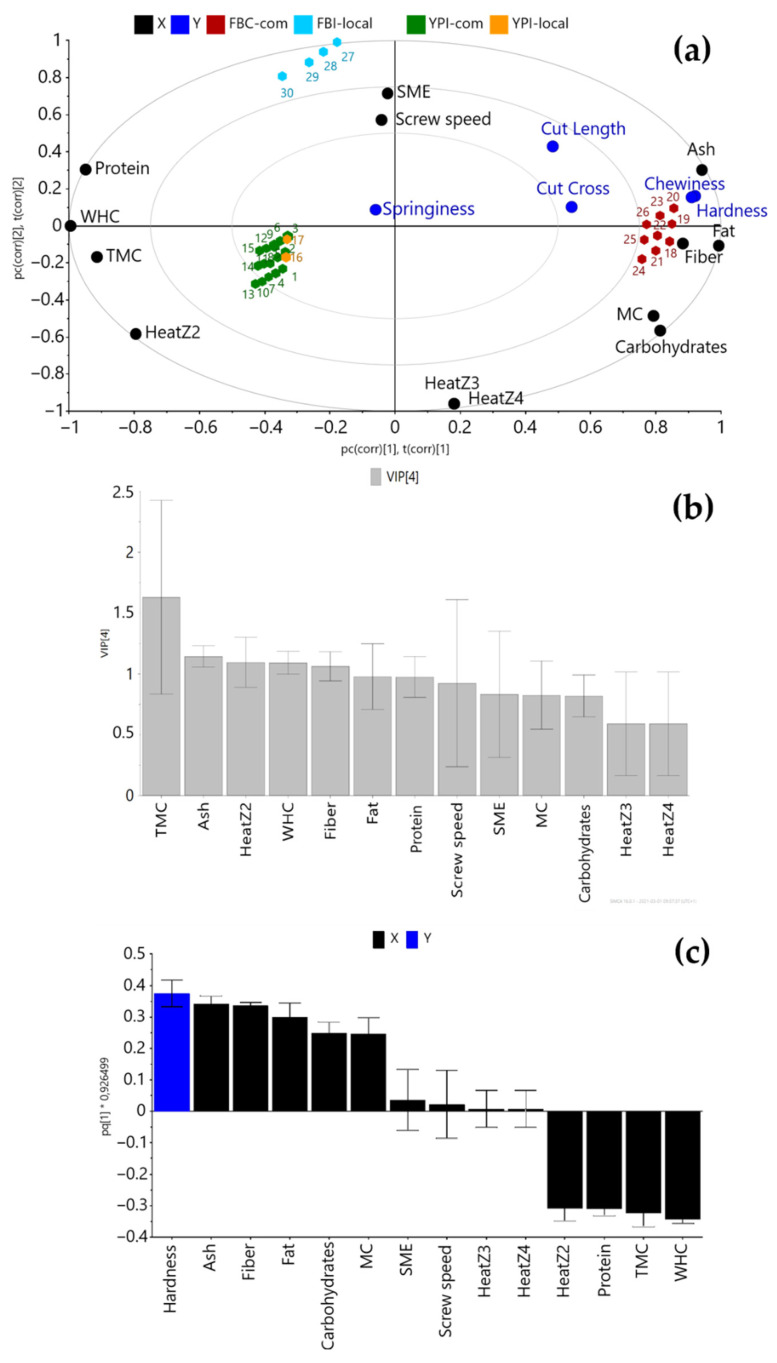
(**a**) Partial least square (PLS) bi-plot showing the correlation between protein isolate/concentrate composition and extrusion parameters and the texture properties of high-moisture meat analogue (HMMA); X = protein isolate/concentrate composition and extrusion parameters; Y = texture properties of HMMA; (**b**) variable of importance (VIP ≥ 1) plot showing the main variables affecting the texture of HMMA; and (**c**) loading plots of the orthogonal partial least squares (OPLS) model for hardness. TMC = target moisture content; WHC = water-holding capacity; MC = moisture content of protein isolate/concentrate; SME = specific mechanical energy; YPI = yellow pea isolate; FBC = faba bean concentrate; FBI = faba bean isolate.

Unsupervised PCA revealed different groupings of HMMA samples based on the raw material (coefficient of determination R2X(cum) = 0.75, coefficient of prediction Q2(cum) = 0.65) (figure not shown). The PLS model, using protein isolate/concentrate composition and extrusion parameters as X-variables and texture properties of HMMA as Y-variables, resulted in a coefficient of determination (R2Y(cum)) of 0.50 and the coefficient of prediction (Q2(cum)) of 0.38 (Figure 4a).

In the PLS bi-plot (Figure 4a), the HMMA samples made from FBC-com and FBI-local were located in separate quadrants, indicating a difference in texture properties compared with HMMA samples made from YPI-com and YPI-local, which were more similar in terms of texture. As indicated by the variable of importance plot (VIP) for the PLS model, the variables that were most important for the separation of the different HMMA samples in the PLS model were target moisture content and temperature in Z2 (Figure 4b). Ash, fiber, fat and protein content and WHC of the protein isolates/concentrate were also important for the texture of the HMMA. For the specific texture parameter hardness, the OPLS model suggested that the hardness of HMMA was positively correlated with ash and fiber content of the protein isolate/concentrate, but negatively correlated with WHC and target moisture content of the protein isolate/concentrate, when adding all four raw materials to the same model (Figure 4c).

#### 3.3.1. Effects of Protein Isolate/Concentrate Composition

As suggested by the PLS and the OPLS models shown in Figure 4, the ash content of the protein isolates/concentrate was an important factor affecting the texture of HMMA, with higher ash content correlated with harder texture. Calcium ions have previously been found to play an essential role in forming a fibrous layered texture in a meat analogue from caseinate mixture, whereas a sodium caseinate mixture did not result in any fibrous structure [40]. Formation of fibrous structure in that study was attributed to the presence of divalent calcium ions causing stronger interactions between the caseinate micelles, resulting in aggregation and better texturization of the meat analogue [37]. Thus, it appears that disulphide bonds are not the only important factor in the formation of the fibrous structure during extrusion, and that there is also a contribution from ionic bonds. Faba bean contains higher amounts of calcium, phosphorus, iron and zinc than yellow pea [41,42], so when faba bean was extruded using the same extrusion parameters and the same target moisture content as yellow pea, the hardness was significantly higher (2578 g) than that found for yellow pea (1760 g) (Appendix C, Table A4). This difference might be due to the higher mineral content in the faba bean isolate/concentrate contributing more ionic bonds, which in turn increased the hardness of HMMA.

The hardness of HMMAs were found to be positively correlated with total dietary fiber and fat content of the protein isolates/concentrate and negatively correlated with the protein content (Figure 4c, Table 1). FBC-com had the lowest protein content of the raw materials investigated, and the corresponding HMMA had the highest values for hardness and chewiness (Appendix C, Table A4). FBC-com was produced using a dry fractionation technique that resulted in lower protein content and higher content of fat, fiber and carbohydrate than the protein isolates investigated (Table 1). Pietsch et al. [43] concluded that when the raw material contains a substantial amount of non-nitrogenous components (such as polysaccharides), the rheological properties of the mix during extrusion and texturization of HMMA are more influenced by protein-polysaccharide interactions than by protein–protein interactions. High extrusion temperature may enhance the compatibility of proteins and polysaccharides, thus reducing the microstructural interface between them and leading to a product with higher rigidity, but lower texturization [13,44,45], as also found in the present study.

Another important factor affecting the texture of HMMA, according to the OPLS model, was the WHC of the protein isolates/concentrate, with lower WHC correlated to higher hardness (Figure 4c). WHC depends on the conformation of proteins, the number of hydrophilic sites and carbohydrate content [46]. Lower WHC might indicate that more proteins are in their native state [36]. However, upon hydration, heating and shearing in the extruder, the native proteins start to unfold, resulting in increased interaction between the proteins and other components, such as polysaccharides. In the present study, FBC-com had significantly lower WHC than the other three isolates investigated. HMMA produced from FBC-com also had a firmer texture in general, which can be partly explained by its low capacity to hold water.

#### 3.3.2. Effect of Extrusion Parameters

The texture properties and cutting strength of HMMAs decreased as the target moisture content increased (Appendix C, Table A4). This is agreement with findings by others of a decrease in hardness, chewiness and cutting strength in HMMA made from soy and lupin protein with increasing moisture content [18,30]. At higher moisture content, the viscosity and temperature of the mix in the barrel decreases, causing incomplete protein denaturation and therefore less protein interaction and cross-linking, leading to a softer product [18,30]. YPI-com and FBC-com required a different target moisture content to produce the meat analogue (Appendix C, Table A4). HMMA samples produced from FBC-com with moisture content above 62% were very compact, with a fudge-like texture and no layered fibers, possibly due to the much lower protein content (55% wb) in FBC-com compared with YPI-com (79% wb) (Table 1). Protein interactions and cross-linking are prerequisites to create the layered fibrous structure of HMMA, and hence a lower moisture content was needed to produce HMMA from FBC-com.

The extrusion temperature in Z2 appeared to be an important variable affecting the texture of HMMA (Figure 4b). The same extrusion temperature in Z2 (60 °C) was used to produce HMMA from FBI-local and FBC-com. HMMA samples produced from both commercial and local faba bean proteins had higher values in all texture properties compared with HMMA made from yellow pea proteins (Appendix C, Table A4). This might be due to differences in protein composition and properties (Appendix B, Figure A2) between faba bean and yellow pea [47].

The effect of different screw speeds on texture properties and cutting strength of HMMA was not consistent (Appendix C, Table A4). It was observed that screw speed affected the hardness and cutting strength to a greater extent at higher moisture contents (69–70%). In this moisture range, higher screw speed led to an increased degree of texturization, and thus higher values of hardness and cutting strength (crosswise and lengthwise). At lower moisture contents (≤68%), there was no clear relationship between screw speed and HMMA texture parameters (Appendix C, Table A4). This finding was supported by the multivariate statistical analysis, where screw speed had an impact on the texture parameters (Figure 4b). However, the error bar was high, indicating that screw speed was an essential factor for some HMMA formulations investigated, but not for others. Zhang et al. [48] found that increased screw speed (from 250 to 350 rpm) led to an increased degree of texturization of vegetable protein from peanut, but that when the screw speed increased further (450 rpm), the strong shear force led to a weaker fibrous structure. Palanisamy et al. [30] found that a higher screw speed led to higher cutting strength of HMMA from lupin protein, due to increased cross-linking and polymerization. These differences between studies may be due to differences in the raw material and extrusion parameters used.

## 4. Conclusions

High-moisture extrusion cooking was successfully applied to produce HMMAs with fibrous layered structures from yellow pea and faba bean proteins, both imported commercial and local. The extruder processing parameter settings had to be adjusted for the different types of protein isolate/concentrate, e.g., a lower target moisture content was needed for faba bean proteins compared with yellow pea proteins. The most important factors affecting the texture properties of HMMAs in this study were the ash, fiber, fat and protein content and water-holding capacity of the protein isolate/concentrate. Studied extrusion parameters affected the texture of HMMA. Further studies on the effect of different screw configurations on the fibrous structure formation could be explored, as well as sensory studies regarding the textural properties and acceptability of the HMMAs.

## Figures and Tables

**Table 1:** Proximate composition (wet basis) and water-holding capacity (WHC) of commercial and local pulse protein isolates/concentrate ^1^.

Parameter	Yellow Pea		Faba Bean	
	Isolate Commercial	Isolate Local	Concentrate Commercial	Isolate Local
Moisture (%)	6 ± 0.2 ^b^	2 ± 0.1 ^c^	9 ± 0.2 ^a^	2 ± 0.3 ^c^
Ash (%)	4 ± 0.1 ^d^	5 ± 0.1 ^c^	6 ± 0.1 ^a^	5 ± 0.1 ^b^
Protein (%)	79 ± 1 ^c^	81 ± 1 ^b^	56 ± 1 ^d^	88 ± 1 ^a^
Fat (%)	0.2 ± 0.0 ^d^	3 ± 0.1 ^b^	3 ± 0.1 ^a^	0.3 ± 0.0 ^c^
Carbohydrate, by difference (%)	9.2 ± 0.7 ^b^	7.6 ± 0.4 ^c^	15 ± 1 ^a^	2.9 ± 0.5 ^d^
Total dietary fibre (%)	1.6 ± 0.1 ^b^	1.6 ± 0.1 ^b^	10 ± 0.4 ^a^	1.3 ± 0.2 ^b^
WHC (mL/g)	3 ± 0.1 ^b^	4 ± 0.1 ^a^	1 ± 0.0 ^d^	3 ± 0.1 ^c^

^1^ All values shown are mean (% wet basis) ± SD (*n* = 3). Different lowercase letters within rows indicate a significant difference (Tukey’s test, *p* < 0.05) between different protein isolates/concentrate.

## Data Availability

All data generated or analyzed during this study are included in this article.

## Appendix B

**Table A1:** Extrusion parameters tested during preliminary trials with commercial yellow pea protein isolate (total mass flow 3 kg/h) to determine the temperature range in extrusion barrel zones 1–4 (Z1–Z4) to be used in experiments.

Target Moisture Content (%)	Screw Speed (rpm)	Processing Temperature Zone (°C)			
		Z1	Z2	Z3	Z4
65	800	40	80	110	110
		40	80	110	130
		40	80	120	140
		40	80	130	150
68	800	40	80	110	110
		40	80	110	130
		40	80	120	140
		40	80	130	150
70	800	40	80	110	110
		40	80	110	130
		40	80	120	140
		40	80	130	150

**Table A2:** Texture properties of high-moisture meat analogue (HMMA) made from commercial yellow pea protein isolate during preliminary extrusion temperature trials ^1^ (total mass flow 3 kg/h; screw speed 800 rpm).

Target Moisture Content (%)	Processing Temperature Zone (°C)				SME ^2^ (kJ/kg)	Texture Properties			Cutting Strength	
	Z1	Z2	Z3	Z4		Hardness (g)	Springiness	Chewiness (g)	Crosswise (g)	Lengthwise (g)
65	40	80	110	110	1179 ± 47 ^a^	3996 ± 273 ^a^	0.9 ± 0.06 ^a^	3408 ± 197 ^a^	2529 ± 191 ^b^	2914 ± 57 ^a^
	40	80	110	130	1152 ± 47 ^ab^	3638 ± 176 ^a^	0.9 ± 0.02 ^a^	3219 ± 153 ^a^	3184 ± 127 ^a^	2731 ± 78 ^a^
	40	80	120	140	1125 ± 81 ^ab^	2199 ± 230 ^b^	0.9 ± 0.05 ^a^	1935 ± 165 ^b^	3378 ± 193 ^a^	2674 ± 79 ^a^
	40	80	130	150	1018 ± 46 ^b^	2003 ± 56 ^b^	0.9 ± 0.05 ^a^	1813 ± 49 ^b^	2977 ± 295 ^ab^	2542 ± 326 ^a^
68	40	80	110	110	1152 ± 123 ^a^	3059 ± 126 ^a^	0.9 ± 0.07 ^a^	2569 ± 85 ^a^	1708 ± 152 ^a^	1597 ± 83 ^b^
	40	80	110	130	1018 ± 46 ^a^	2092 ± 114 ^b^	0.9 ± 0.02 ^a^	1856 ± 132 ^b^	1842 ± 130 ^a^	1951 ± 178 ^a^
	40	80	120	140	992 ± 46 ^a^	2760 ± 159 ^a^	0.9 ± 0.07 ^a^	2364 ± 86 ^a^	1811 ± 37 ^a^	1547 ± 114 ^b^
	40	80	130	150	992 ± 46 ^a^	1428 ± 88 ^c^	0.9 ± 0.02 ^a^	1261 ± 69 ^c^	1740 ± 193 ^a^	1744 ± 61 ^ab^
70	40	80	110	110	1259 ± 46 ^a^	2835 ± 143 ^a^	0.9 ± 0.07 ^a^	2393 ± 122 ^a^	1486 ± 72 ^a^	1494 ± 54 ^a^
	40	80	110	130	1179 ± 47 ^a^	2495 ± 253 ^a^	0.9 ± 0.05 ^a^	2093 ± 207 ^a^	1514 ± 131 ^a^	1553 ± 18 ^a^
	40	80	120	140	1072 ± 46 ^b^	2407 ± 227 ^a^	0.8 ± 0.04 ^a^	2035 ± 211 ^a^	1797 ± 51 ^a^	1621 ± 149 ^a^
	40	80	130	150	992 ± 46 ^c^	1795 ± 86 ^b^	0.9 ± 0.07 ^a^	1599 ± 105 ^b^	2386 ± 269 ^b^	1091 ± 27 ^b^

^1^ All values shown are mean ± SD (n = 3). ^2^ Specific mechanical energy. Means with different lowercase letters within columns for each target moisture content are significantly different (Tukey’s test, *p* < 0.05).

**Table A3:** Target moisture content and screw speed values tested in preliminary trials after extrusion temperature determination (total mass flow 2 kg/h).

Raw Material	Processing Temperature Zone (°C)				Target Moisture Content (%)	Screw Speed (rpm)
	Z1	Z2	Z3	Z4		
Yellow pea isolate commercial (YPI-com)	40	80	130	150	66	400
						600
						800
					67	400
	40	80	130	150		600
						800
					68	400
	40	80	130	150		600
						800
					69	400
	40	80	130	150		600
						800
					70	400
	40	80	130	150		600
						800
Yellow pea isolate local (YPI-local)	40	80	130	150	67	400
						600
						800
Faba bean concentrate commercial (FBC-com)	40	60	130	150	58	400
						600
						800
	40	60	130	150	60	400
						600
						800
	40	60	130	150	62	400
						600
						800
Faba bean isolate local (FBI-local)	40	60	110	130	62	800
					64	800
					66	800
					68	800

## Appendix C

**Table A4:** Effect of extrusion temperature, screw speed and target moisture content on the textural properties of high-moisture meat analogues (HMMAs) produced from different pulse protein isolates/concentrate ^1^ (total mass flow 2 kg/h).

ID	Target Moisture Content (%)	Screw Speed (rpm)	SME ^3^ (kJ/kg)	Texture Profile Analysis ^2^			Cutting Strength ^2^	
				Hardness (g)	Springiness	Chewiness (g)	Crosswise (g)	Lengthwise (g)
Yellow pea isolate commercial (YPI-com) ^1^								
1	66%	400	583 ± 35 ^d^	2229 ± 237 ^e^	0.9 ± 0.09 ^ab^	1950 ± 167 ^fg^	2402 ± 3 ^bcd^	2001 ± 107 ^d^
2		600	1085 ± 91 ^c^	2534 ± 250 ^e^	1.0 ± 0.01 ^a^	2201 ± 237 ^f^	2297 ± 43 ^cd^	2053 ± 308 ^d^
3		800	1527 ± 69 ^a^	2682 ± 168 ^e^	0.9 ± 0.04 ^ab^	2354 ± 147 ^f^	2508 ± 187 ^bc^	1545 ± 221 ^e^
4	67%	400	583 ± 35 ^d^	2389 ± 123 ^e^	0.8 ± 0.04 ^ab^	2021 ± 164 ^fg^	2237 ± 47 ^cd^	1749 ± 55 ^de^
5		600	995 ± 91 ^c^	1824 ± 147 ^efg^	0.9 ± 0.02 ^ab^	1570 ± 94 ^hi^	1427 ± 70 ^efg^	1466 ± 163 ^ef^
6		800	1447 ± 121 ^a^	2131 ± 139 ^efg^	0.9 ± 0.02 ^ab^	1818 ± 103 ^gh^	1623 ± 59 ^ef^	1260 ± 71 ^f^
7	68%	400	563 ± 35 ^d^	1986 ± 133 ^efg^	0.9± 0.02 ^ab^	1986 ± 133 ^fg^	2091 ± 105 ^de^	1706 ± 87 ^de^
8		600	904 ± 1 ^c^	1774 ± 105 ^fg^	1.0 ± 0.01 ^a^	1544 ± 80 ^hi^	1756 ± 102 ^def^	1169 ± 93 ^f^
9		800	1487 ± 69 ^a^	1760 ± 114 ^fg^	0.9 ± 0.09 ^ab^	1524 ± 75 ^hi^	1657 ± 74 ^ef^	1326 ± 83 ^f^
10	69%	400	563 ± 35 ^d^	1930 ± 67 ^efg^	0.9± 0.04 ^ab^	1642 ± 45 ^gh^	1818 ± 44 ^def^	1674 ± 29 ^def^
11		600	1085 ± 91 ^c^	2016 ± 133 ^efg^	1.0 ± 0.01 ^a^	1785 ± 107 ^gh^	1833 ± 71 ^de^	2011 ± 216 ^d^
12		800	1487 ± 69 ^a^	2433 ± 27 ^e^	0.9 ± 0.06 ^ab^	2075 ± 18 ^fg^	1969 ± 160 ^de^	2112 ± 204 ^d^
13	70%	400	643 ± 35 ^d^	1493 ± 153 ^g^	0.9± 0.05 ^ab^	1259 ± 119 ^hi^	930 ± 28 ^h^	702 ± 132 ^g^
14		600	1176 ± 91 ^c^	1702 ± 84 ^fg^	0.9 ± 0.02 ^ab^	1446 ± 98 ^hi^	1270 ± 164 ^g^	1277 ± 47 ^f^
15		800	1567 ± 121 ^a^	2426 ± 101 ^e^	0.9 ± 0.06 ^ab^	2028 ± 31 ^fg^	1599 ± 16 ^efg^	1325 ± 74 ^f^
Yellow pea isolate local (YPI-local) ^1^								
16	67%	400	502 ± 35 ^d^	2177 ± 175 ^efg^	0.9 ± 0.09 ^ab^	1805 ± 176 ^gh^	1846 ± 140 ^def^	1451 ± 220 ^f^
17		600	1025 ± 52 ^c^	2252 ± 210 ^ef^	0.9 ± 0.09 ^ab^	1796 ± 148 ^gh^	1828 ± 275 ^def^	1780 ± 32 ^def^
		800						
Faba bean concentrate commercial (FBC-com) ^1^								
18	58%	400	643 ± 35 ^d^	6136 ± 190 ^a^	0.9 ± 0.01 ^ab^	5185 ± 174 ^a^	3149 ± 94 ^a^	2730 ± 71 ^c^
19		600	1146 ± 53 ^c^	6120 ± 245 ^a^	0.9 ± 0.06 ^ab^	5264 ± 254 ^a^	3041 ± 45 ^a^	2679 ± 159 ^cd^
20		800	1567 ± 121 ^b^	4637 ± 325 ^c^	0.9 ± 0.08 ^ab^	4039 ± 244 ^c^	2817 ± 32 ^ab^	2293 ± 94 ^d^
21	60%	400	603 ± 60 ^d^	5475 ± 225 ^b^	0.9 ± 0.04 ^ab^	4806 ± 187 ^a^	3107 ± 157 ^a^	2659 ± 74 ^cd^
22		600	995 ± 2 ^c^	4496 ± 185 ^c^	0.9 ± 0.05 ^ab^	3914 ± 188 ^bc^	2336 ± 97 ^cd^	2212 ± 99 ^d^
23		800	1607 ± 70 ^b^	5222 ± 241 ^b^	0.9 ± 0.02 ^ab^	4420 ± 240 ^ab^	2570 ± 183 ^bc^	2317 ± 142 ^cd^
24	62%	400	583 ± 35 ^d^	3780 ± 160 ^d^	0.9 ± 0.01 ^ab^	3002 ± 222 ^d^	1520 ± 51 ^fg^	1644 ± 61 ^ef^
25		600	1146 ± 53 ^c^	4522 ± 74 ^c^	0.9 ± 0.01 ^ab^	3631 ± 21 ^cd^	1697 ± 27 ^def^	1813 ± 61 ^de^
26		800	1567 ± 121 ^b^	4009 ± 93 ^cd^	0.9 ± 0.07 ^ab^	3339 ± 90 ^d^	1788 ± 44 ^def^	1547 ± 14 ^ef^
Faba bean isolate local (FBI-local) ^1^								
27	62%	800	1889 ± 121 ^a^	2648 ± 188 ^e^	0.9 ± 0.02 ^ab^	2309 ± 152 ^f^	2423 ± 109 ^cd^	3397 ± 180 ^ab^
28	64%	800	1809 ± 70 ^ab^	3301 ± 274 ^d^	0.9 ± 0.05 ^ab^	2808 ± 216 ^e^	2249 ± 184 ^cd^	2479 ± 136 ^cd^
29	66%	800	1728 ± 121 ^ab^	3524 ± 150 ^d^	0.9 ± 0.09 ^ab^	2992 ± 133 ^de^	2019 ± 92 ^de^	1933 ± 29 ^de^
30	70%	800	1809 ± 69 ^ab^	2578 ± 26 ^e^	0.9 ± 0.08 ^ab^	2128 ± 48 ^fg^	1204 ± 146 ^g^	1553 ± 102 ^ef^
References								
31	Commercial soybean HMMA			500 ± 45 ^h^	0.9 ± 0.08 ^ab^	408 ± 39 ^j^	1204 ± 119 ^g^	1553 ± 52 ^ef^
32	Boiled chicken			2332 ± 148 ^e^	0.8 ± 0.09 ^b^	1679 ± 86 ^gh^	2102 ± 131 ^d^	3207 ± 227 ^b^
33	Boiled beef			1645 ± 238 ^g^	0.9 ± 0.12 ^ab^	1087 ± 113 ^i^	1821 ± 383 ^ef^	3743 ± 123 ^a^

^1^ Extrusion temperature zone (Z1-Z2-Z3-Z4): YPI-com and YPI-local (40-80-130-150 °C), FBC-com (40-60-130-150 °C), FBI-local (40-60-110-130 °C). ^2^ All values shown are mean ± SD (*n* = 3). ^3^ Specific mechanical energy. Means with different lowercase letters within columns for each texture parameter are significantly different (Tukey’s test, *p* < 0.05).
